# Autonomic dysfunction in patients with episodic cluster headache during remission period

**DOI:** 10.1007/s13760-025-02915-8

**Published:** 2025-10-13

**Authors:** Alba López-Bravo, Elena Bellosta Diago, Marisa de la Rica Escuín, Laura Díez Galán, Sonia Santos Lasaosa

**Affiliations:** 1 Neurology Department, Reina Sofía Hospital, Tudela, Navarra Spain; 2 Aragon Health Research Institute, Zaragoza, Spain; 3 Department of Health Sciences, Public University of Navarra, Tudela, Spain; 4 Neurology Department, Clínico Universitario Lozano Blesa Hospital, Zaragoza, Spain

**Keywords:** Autonomic dysfunction, Autonomic nervous system, Cluster headache, Headache, Norepinephrine, Parasympathetic, Sympathetic

## Abstract

**Background:**

The hypothalamus is involved in cluster headache (CH) pathophysiology and is a hub for autonomic control. While cranial autonomic symptoms are prominent during attacks, other autonomic manifestations may be present in CH. This study aims to explore the autonomic nervous system (ANS) in patients with CH during remission period.

**Methods:**

Cross-sectional study including 30 CH and 30 age- and sex-matched controls. We analysed time- and frequency-domain parameters of heart rate variability (HRV) and active orthostatic tests. To investigate the sympathetic nervous system, plasma norepinephrine (NE) levels were determined. All assessments were performed during remission period.

**Results:**

All HRV parameters were lower in CH; the percentage of adjacent R-R intervals that differ by more than 50 milliseconds (pNN50) and standard deviation of normal-to-normal R-R intervals in 24 h (SDNN) were significantly lower in CH (pNN50, 31.0 [5.3–44.3] vs. 44.5 [25.8–58.5], *p* = 0.043; SDNN, 79.6 ± 42.6 vs. 99.6 ± 42.6, *p* = 0.004). All other time-domain parameters, including the root mean square of successive R-R differences (RMSSD) were lower in CH than in controls (RMSSD 59.5 ± 36.9 vs. 77.3 ± 39.4, *p* = 0.077). Compared to controls, mean HR was significantly higher in CH (64.2 [59.6–75.8] vs. 60.4 [57.3–62.7], *p* = 0.038). Supine and upright NE levels were significantly higher in CH, (supine 228.9 [161.6-324.1] vs. 209.9 [151.2-314.1], *p* = 0.015; standing 376.1 [264.6-527.8 vs. 327.4 [256.4-400.9], *p* = 0.019).

**Conclusions:**

The present study indicates a significant decrease in HRV and an upward trend of plasmatic NE levels in CH during remission periods, suggesting an imbalance of the ANS in this state.

**Graphical Abstract:**

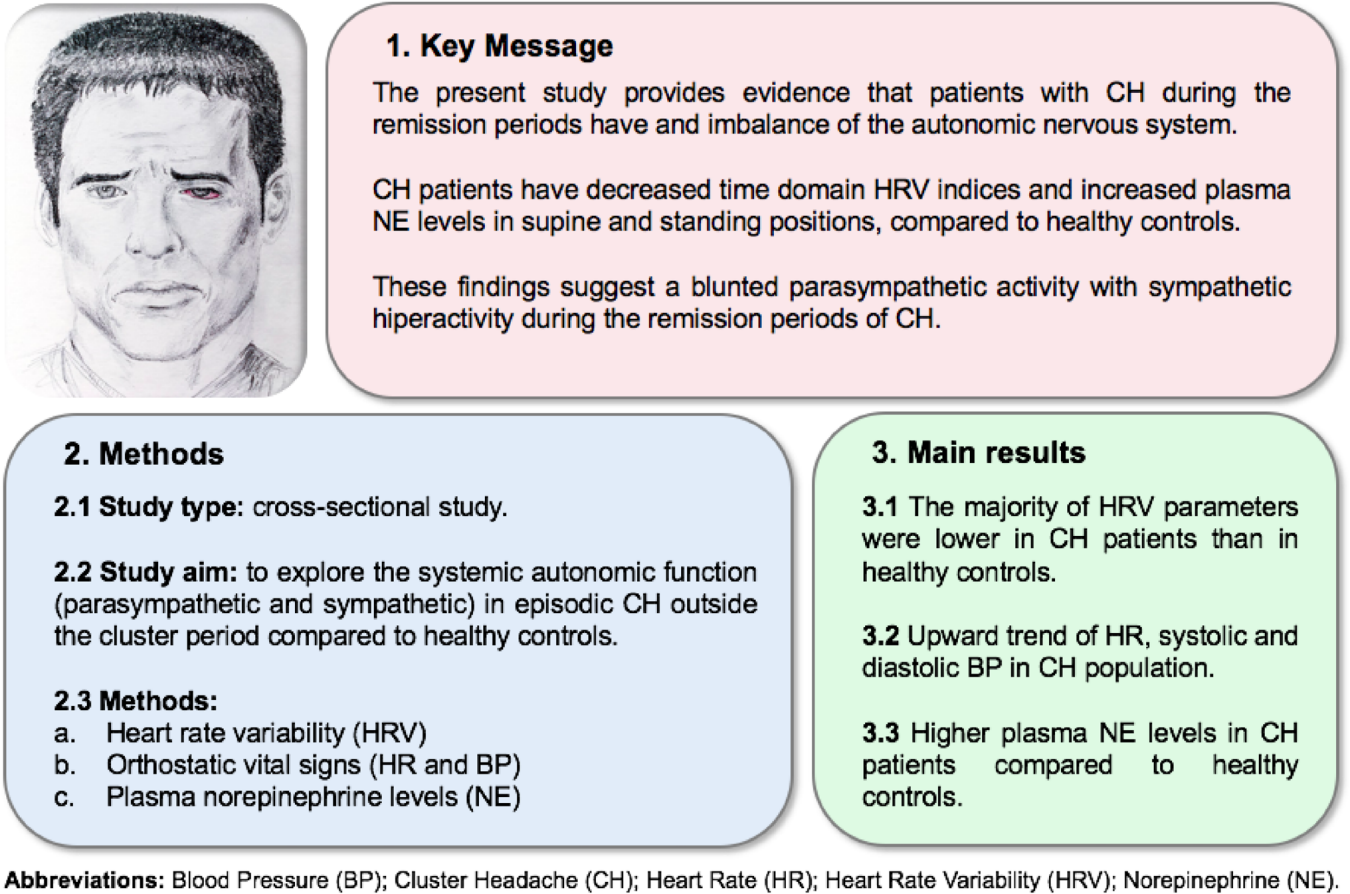

## Introduction

Cluster headache (CH) is a trigeminal autonomic primary headache characterized by unilateral pain attacks accompanied by cranial autonomic symptoms. Most patients with CH have bouts with daily headache attacks interspersed with symptom free periods (remission) that last for months to years at a time. This is the episodic CH phenotype (ECH), in contrast to chronic CH (CCH), which is defined by remission periods lasting less than three months per year [1].

The pathophysiology of CH is still not known, but findings from functional neuroimaging studies have suggested a central role of the posterior hypothalamus in the genesis of attacks [2]. However, other brain regions, known to be part of the pain processing brain network, are also thought to be involved in the pathogenesis of CH [3]. Cortical-hypothalamic-brainstem functional interconnections that can switch between out-of-bout and in-bout periods, igniting the trigeminovascular system and the consensual trigeminal autonomic reflexes, may represent the “neuronal background” of CH [3].

Trigeminal autonomic reflex activation is the basis for cranial autonomic symptoms (CAS) experienced during CH attacks [4]. In addition to CAS, systemic autonomic dysfunction may be present in CH [5, 6], but studies have revealed inconsistent findings. A study assessed cardiovascular autonomic function at different stages of the disease (during and outside a cluster period) revealed no significant differences, except for resting diastolic blood pressure (BP), which was higher during the cluster period [5]. In this regard, increased BP and a blunted autonomic response to head-up tilt table test have been described in CH patients during the cluster period compared to healthy controls [6]. Less standardized studies have investigated autonomic function in the remission phase of episodic CH. Ofte et al.., studied cranial autonomic function during the remission phase in CH patients underwent dynamic pupillometry. They found a significant attenuation of pupillary light reflexes in both eyes compared to healthy controls, suggesting a reduction of cranial parasympathetic tone in the pain- free state of CH [7].

In addition, other authors have evaluated the potential role of different neurotransmitters and neuromodulators in the pathogenesis of ECH and CCH. As summarized in Table 1, these studies have demonstrated an involvement of the hypothalamus in the genesis of CH and autonomic nervous system (ANS) dysregulation [8–14]. However, clear conclusions could not be made from data gathered and further studies with standardized tests are needed to elucidate the relationship of systemic autonomic function and CH.

**Table 1:** Circulating amines and traces levels in controls and patients with cluster headache (CH)

Study	Case numbers (control/CH)	Type of CH (ECH/CCH)	Sample	Phase (remission/cluster/attack)	Finding	Interpretation
Igarashi et al. (1985) [8]	33/20	ECH	Plasma	Remission period (*not specified*) Spontaneous and provoked attack by nitroglycerin	NE n.s. ↑ NE	Involvement of the SNS in the pathogenesis of the CH attack
D´Andrea et al. (1992) [9]	15/23 15/23 15/14	ECH	Platelet	Remission period (*not specified*) Cluster period Painful attack	↓ NE ↓ NE ↓ NE	Biochemical evidence of SNS hypofunction in CH
Strittmatter et al. (1996) [10]	15*/12	ECH	Plasma/cerebrospinal fluid	Cluster period (*last attack at least 12 h previously*)	↓ NE	Reduced activity of the SNS during the cluster period in CH Involvement of the hypothalamus, and autonomic nervous system in the genesis of CH
D´Andrea et al. (2004) [11]	36/44	ECH	Plasma/platelet	Remission period Cluster period	↑ Tyramine ↑ Octopamine ↑ Synephrine	Sympathetic or hypothalamic dysfunction in CH
Meyer et al. (2007) [12]	10/9	ECH	Plasma**	Remission period (*not specified*)	NE n.s. ↑ GH	Permanent hypothalamic disturbance
D´Andrea et al. (2017) [13]	16/23	CCH	Plasma	Cluster period	↑ NE ↑ Tyramine ↑ Dopamine ↓ Octopamine ↓ Synephrine	Anomalies in tyrosine metabolism constitute a predisposing factor for the chronification of ECH
D´Andrea et al. (2017) [14]	28/23	CCH	Plasma	Cluster period	↑ NE ↑ E ↑ Tryptamine	Activation of endothelial TAAR1 receptors followed by the release of nitric oxide Role of tryptamine in the pathogenesis of CCH

**Abbreviations**: CH, cluster headache; ECH, episodic CH; CCH, chronic CH; GH, growth hormone; E, epinephrine; NE, norepinephrine; SNS, sympathetic nervous system; TAAR, trace amine-associated receptor

n.s. denotes non-significant

*All controls suffered from neuromuscular diseases **Nocturnal secretion of NE

While CH are innately connected with the ANS, results of autonomic testing in previous studies have been variable in this disorder, especially, in the remission period. In this context, we hypothesized that patients with CH might experience ANS dysfunction, particularly during remission period. Accordingly, the overall aim of this study was to explore systemic autonomic function (sympathetic and parasympathetic) in ECH patients outside the cluster period compared to controls, using standardized autonomic assessment tests.

## Methods

### Design and participants

This cross-sectional study recruited thirty consecutive patients with ECH from the Headache Unit of the Hospital Clínico Universitario Lozano Blesa (Zaragoza, Spain) between September 2019 and May 2020. Inclusion criteria were (1) age 18–65 years (2) diagnosis of ECH according to the International Classification of Headache Disorders, 3rd edition (ICHD-3) [1] with the last cluster period at least 12 weeks before (3) without prophylactic headache medication at the time of recruitment (4) absence of pharmacological treatment of any kind in the month prior to the study, with the exception of non- steroidal anti-inflammatory drugs (free of symptomatic treatment in the previous 24 h).

### Exclusion criteria

All subjects with conditions known to affect ANS regulation were excluded, including: cardiac disorders, poorly-controlled hypertension or postural hypotension, chronic obstructive pulmonary disease, kidney and liver disease, endocrinological disorders and malnutrition, drug abuse (except for nicotine dependence) or chronic medications (potential effect on BP/HR. Subjects with signs of any other neurological disorder, particularly peripheral neuropathy, and serious psychiatric cognitive disorders, were also excluded. The control group was recruited during the same period and consisted of thirty subjects matched for age and sex with CH patients. All controls were interviewed to ensure they were healthy and free of neurological disorders, including other types of headache.

The study was approved by the Regional Research Ethics Committee of Aragon (CEICA) and complied the scientific and ethical guidelines for human research established by the Helsinki Accord. All subjects provided written informed consent to participate in this study.

### Protocol

**Fig. 1:**
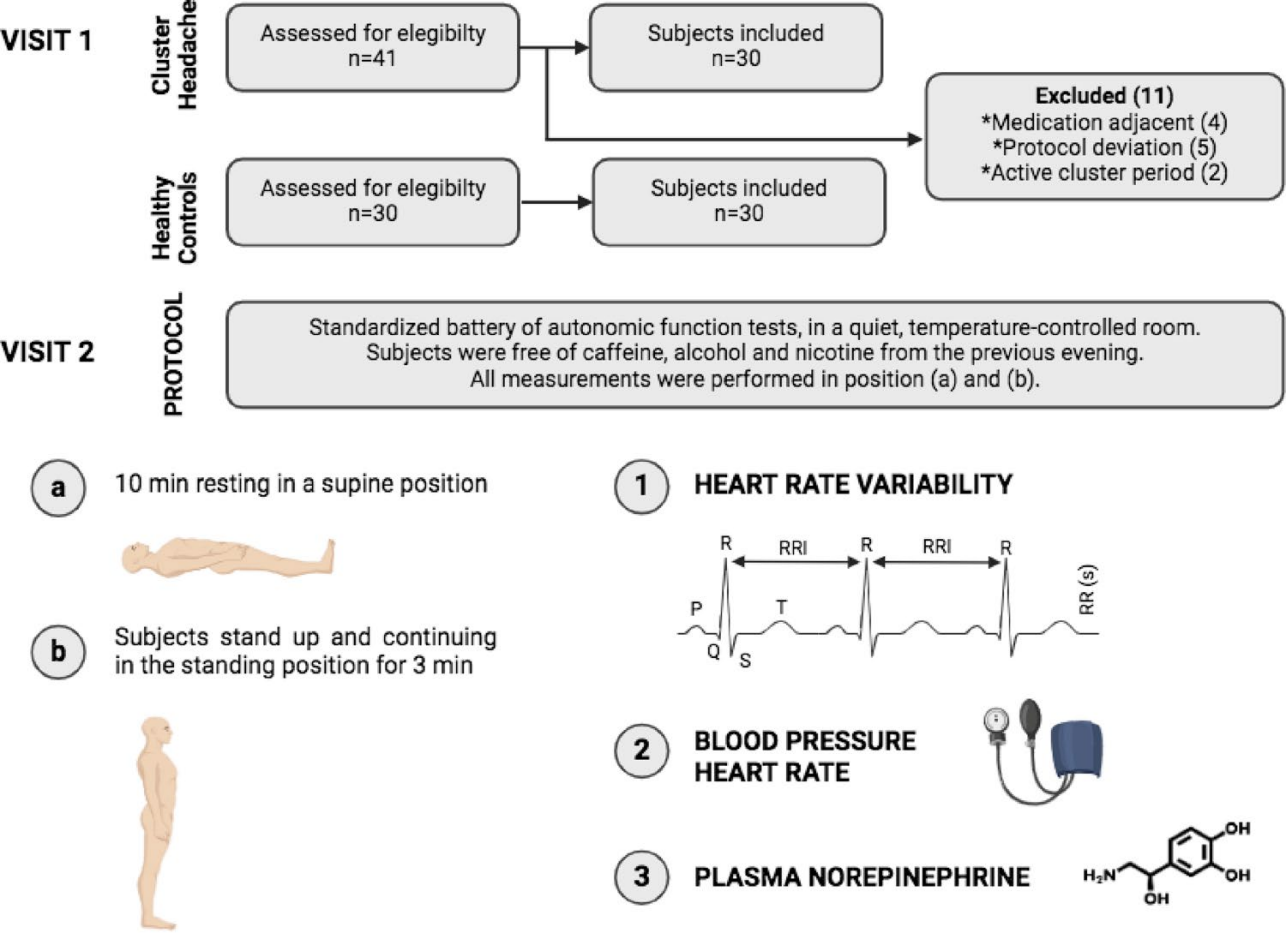
Study flow diagram and protocol

All subjects were free of caffeine, alcohol, nicotine and medication from the previous evening. All procedures were performed in the morning (8–10 am), after 10 min of rest and avoiding previous physical activity. Subjects underwent a comprehensive medical history (*exclusion criteria*, subjects with previous disorders were excluded) and a standardized battery of autonomic function tests, in a quiet, temperature-controlled room (Fig. 1). Three researchers (ALB, MRE, LDG) who were blinded to clinical data performed the battery of standardized autonomic test.

### Blood pressure (BP) and heart rate variability (HRV)

Baroreflex-mediated withdrawal of cardiac parasympathetic activity and sympathetic activation maintain standing BP in healthy persons. Under normal circumstances, systolic BP (SBP) decreases by 10 mmHg, while diastolic BP (DBP) increases by 5 mmHg upon standing from a sitting or supine position. Similarly, HR increases by 5–20 beats per minute. Orthostatic hypotension is caused by a sustained fall in SBP or DBP after standing for 3 min. In contrast, some patients have a paradoxical increase in upright BP to hypertensive levels, presumably due to sympathetic activation overshoot.

- BP and HR were measured with a validated automated cuff sphygmomanometer over the brachial artery on the nondominant arm, using adequate cuffs according to arm circumference. After the subjects had rested for 10 min, three consecutive measures were taken after 1, 2 and 3 min of standing.

HRV is the variation in consecutive heartbeats [R–R interval (RRI)] and is a marker of ANS activity in which increased HRV reflects parasympathetic predominance, whereas decreased HRV suggests sympathetic predominance. HRV were analysed within two domains: time-domain and frequency-domain measures.

The following time-domain parameters were measured [15, 16]:

- Mean RR (ms): the mean of the RRI.
- SDNN (ms): the standard deviation of normal-to-normal RRI. SDNN reflects the parasympathetic component of the autonomic function.
- RMSSD (ms): the root means square of difference between successive normal intervals. It is an important indicator of parasympathetic activity.
- pNN50 (%): the percentage of differences greater than 50 ms between successive normal RRI. It predominantly reflects the parasympathetic activity.

The following frequency-domain parameters were measured [15, 16]:

- LF (ms^2^): it consists of a combination of sympathetic and parasympathetic effects.
- HF (ms^2^): it is considered that is modulated by the parasympathetic activity of ANS.
- LF/HF ratio: the ratio of LF-to-HF power. It reflects the sympathovagal balance and can be used to estimate HRV in general.

HRV assessment was based on RRI records at rest and during during deep paced breathing, collected with a free smartphone app (Elite HRV Inc, version 5.5.6) for Apple via Bluetooth 4.0, and a wireless transmitter Polar H7 (Polar Electro Oy, Kempele, Finland) placed on the patient’s chest [17]. Subjects were trained to breathe at 6 cycles per minute, and respiratory sinus arrhythmia during deep-paced breathing was calculated from the mean of the 3 longest RRI during expiration divided by the mean of the 3 shortest RRI during inspiration (i.e., expiratory: inspiratory [E: I] ratio) [18]. The RRI series obtained were subjected to frequency and time-domain analysis.

### Norepinephrine (NE)

NE is the principal neurotransmitter of the sympathetic nervous system (SNS). NE in the bloodstream emanates mainly from networks of sympathetic nerves that enmesh blood vessels. Considering the sympathoneural origin of NE, plasma NE levels are used to indicate activity of the sympathetic noradrenergic system.

- NE plasma levels: an intravenous catheter was inserted in the forearm into the antecubital vein and venous blood samples were collected through the indwelling catheter after at least 15 minutes’ and after 10 min standing up. The analysis was performed using high- performance liquid chromatography. In addition, patients underwent a complete blood count and metabolic panel to rule out anemia, dehydration or electrolyte imbalances.

### Statistical analysis

First, the data were tested for normality using the Shapiro-Wilk and Kolmogorov-Smirnov tests. For parametric variables, data were expressed as means with standard deviations (SD), and for non-parametric variables, median and interquartile range (IQR). In bivariate analyses, Student’s t-test, one-way ANOVA and Mann-Whitney tests were used to compare variables between groups. Repeated-measures ANOVA was performed to compare the effect of headache characteristics (case-control) and time on levels of NE. For all tests, a two-sided P-value of < 0.05 was used to determine statistical significance. All analyses were performed with R version 4.0.5 (R Foundation for Statistical Computing, Viena, Austria).

## Results

Thirty episodic CH patients and age- and sex-matched controls were included in the analysis. Baseline and clinical characteristics of participants are summarized in Table 2. Demographic characteristics including age, gender, smoking habits, and history of cardiovascular risk factors, were not significantly different between patients with CH and individuals in the control group. Attack and cluster period characteristics were recorded for all patients in the CH group. All included patients were out-of-bout, with a median since the last cluster period of 18.0 months [range, 3.0–48.0].

**Table 2:** Demographic and clinical of participants

Variable		Healthy controls (*n* = 30)	Cluster headache (*n* = 30)
Male, n (%)		30 (100%)	30 (100%)
Age, years ± SD		48.3 ± 8.2	49.5 ± 8.2
Systolic blood pressure*, mmHg ± SD		135.4 ± 16.7	133.5 ± 16.9
Diastolic blood pressure*, mmHg ± SD		81.8 ± 9.9	83.4 ± 10.1
Heart rate*, bpm ± SD		60.7 ± 9.5	65.5 ± 13.7
Age of disease onset, years, [median, IQR]		NA	31.0 [19.0–42.0]
Disease duration, years, [median, IQR]		NA	14.0 [8.0–29.0]
Daily attack frequency** [median, IQR]		NA	2.0 [1.0–2.0]
Daily attack duration, min [median, IQR] Number of attacks per year, [median, IQR]		NA NA	75.0 [48.8–157.5.8.5] 1.0 [1.0–2.0]
Cluster period duration, months [median, IQR] Last bout, months [median, IQR, range]		NA NA	1.0 [1-0-2.8] 18.0 [5.0–24.0, 3.0–48.0]
Laterality, n (%) - Left - Right		NA	18 (60) 12 (40)
Autonomic symptoms, n (%) - Lacrimation - Conjunctival injection - Nasal congestion/rhinorrhoea - Eyelid edema - Forehead and facial sweating - Ptosis - Miosis Smokers, n (%)		NA 8 (26.7)	18 (60) 17 (56.7) 21 (70) 10 (33.3) 11 (36.7) 23 (76.7) 10 (33.3) 23 (76.7)
Hypertension, n (%)		4 (13.3)	5 (16.7)
Dyslipidemia, n (%)		3 (10.0)	4 (13.3)
Diabetes Mellitus, n (%)		0 (0.0)	0 (0.0)
BMI, kg/m^2^, [median, IQR]	23.8 [21.6–28.8]		24.4 [22.1–29.2]

**Notes**: *Blood pressure and heart rate measurements were taken at rest **The daily attack frequency is based on the attack frequency during the peak phase

### Orthostatic heart rate (HR) and blood pressure (BP) changes

**Fig. 2:**
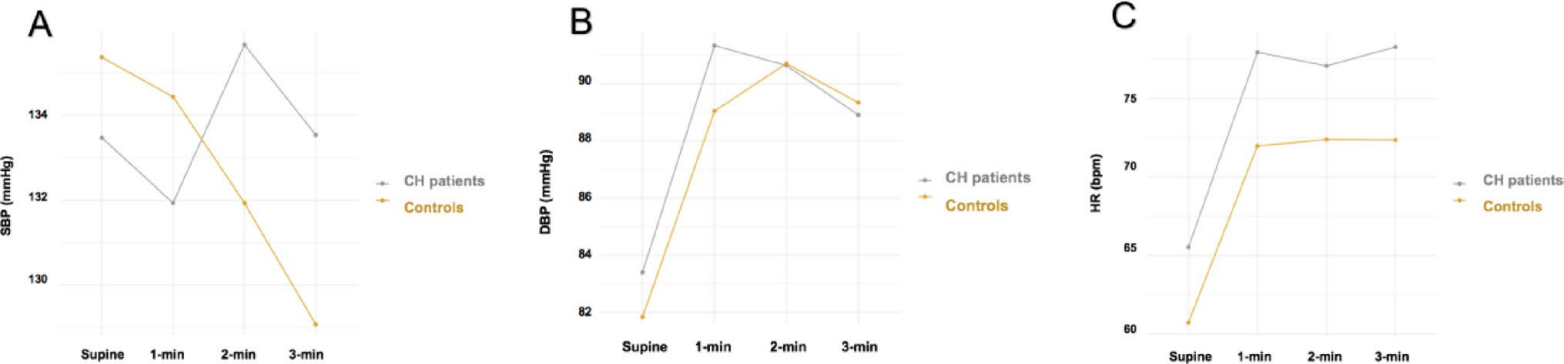
Orthostatic heart rate and blood pressure changes in cluster headache (CH) in remission and control group

Adjusting for baseline values, we observed that absolute HR was higher in CH patients in all four states (supine, 1–2-3-min standing up). In turn, the orthostatic HR increase at each of the measurements was more pronounced in patients with CH compared to healthy controls (Fig. 2). There were no significant differences between groups in supine SBP and responses to 1-minute standing (131.93 ± 12.98 for CH vs. 134.43 ± 14.05 for controls, *p* = 0.477). There was a non-significant increase in 2-minutes and 3-minutes standing up SBP in CH group (135.67 ± 14.49 and 133.53 ± 14.46, respectively), which was not observed in the headache-free group, which had a downward trend in SBP over time. DBP was higher in patients with CH in supine and 1 min standing up, with similar absolute values at minute 3 (Table 3).

**Table 3:** Orthostatic heart rate and blood pressure changes in cluster headache patients and controls

	Cluster headache (*n* = 30)	Healthy controls (*n* = 30)	Differences *p*
SBP supine, mmHg	133.47 (16.85)	135.37 (16.69)	0.662
SBP 1-min standing up, mmHg	131.93 (12.98)	134.43 (14.05)	0.477
SBP 2-min standing up, mmHg	135.67 (14.49)	131.93 (13.77)	0.310
SBP 3-min standing up, mmHg	133.53 (14.46)	129.07 (14.53)	0.238
DBP supine, mmHg	83.40 (10.10)	81.83 (9.93)	0.547
DBP 1-min standing up, mmHg	91.33 (8.35)	89.03 (9.31)	0.318
DBP 2-min standing up, mmHg	90.63 (8.22)	90.70 (12.07)	0.980
DBP 3-min standing up, mmHg	88.90 (9.21)	89.33 (9.94)	0.862
HR supine, bpm	65.53 (13.72)	60.73 (9.49)	0.121
HR 1-min standing up, bpm	77.93 (14.68)	71.97 (10.96)	0.080
HR 2-min standing up, bpm	77.07 (13.20)	72.37 (10.21)	0.129
HR 3-min standing up, bpm	78.27 (14.19)	72.33 (11.17)	0.077

**Notes**: Values are presented as mean and standard deviation (SD). All participants were in normal sinus rhythm

**Abbreviations**: bpm, beats per minute; DBP, diastolic blood pressure; HR, heart rate; SBP, systolic blood pressure

### Heart rate variability analysis (HRV)

All HR parameters, including minimum, maximum and average HR, presented an upward trend in CH, highlighting a significantly higher average HR in the out-of-bout cluster period compared to controls (64.2 [59.6–75.8] vs. 60.4 [57.3–62.67.3.67], *p* = 0.038).

**Fig. 3:**
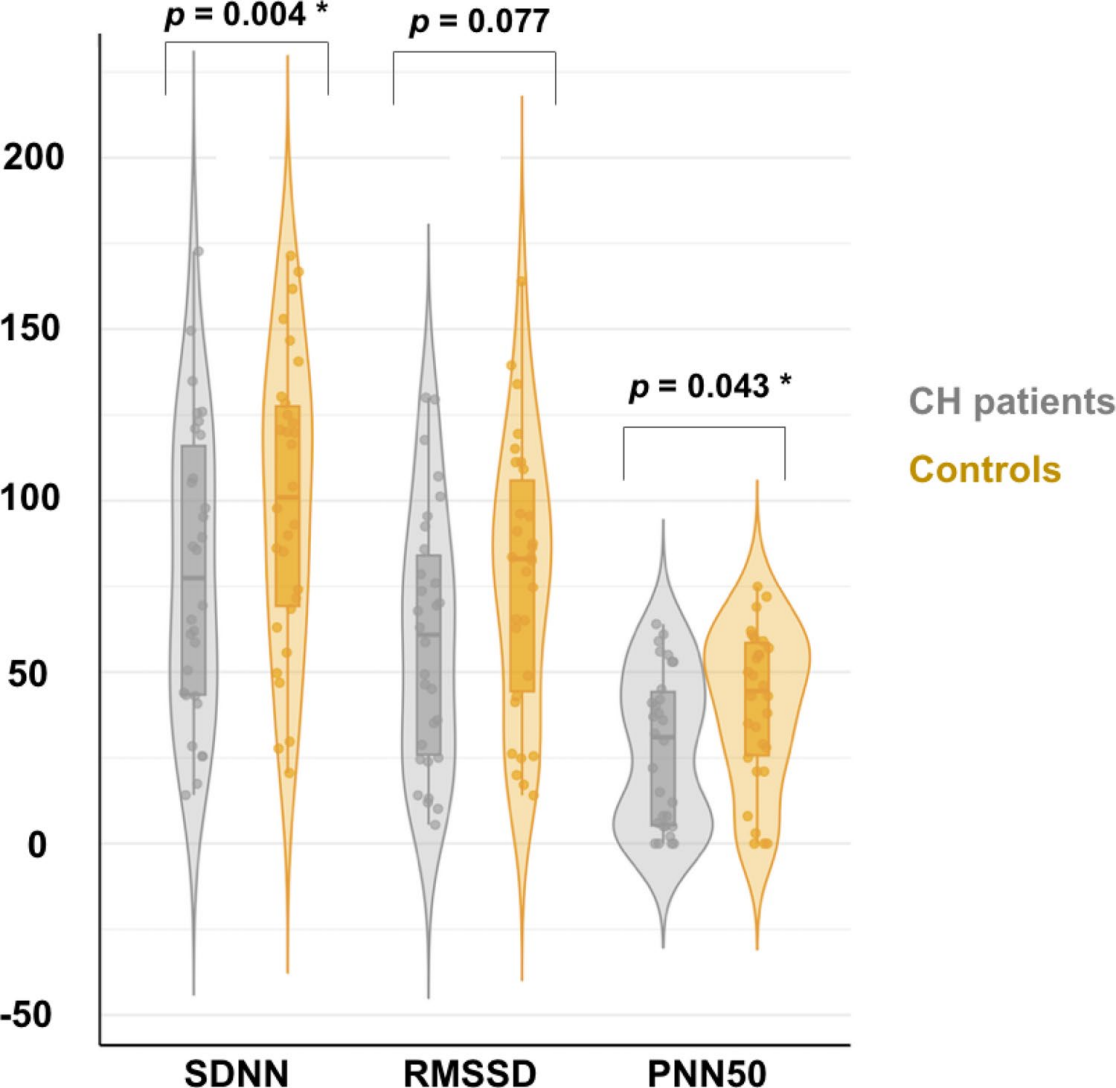
Comparison of time-domain HRV parameters among cluster headache (CH) in remission and control group

Likewise, some time-domain and frequency-domain HRV parameters showed clinically relevant differences between groups. Compared to controls, CH patients had significantly lower pNN50 and SDNN values (pNN50, 31.0 [5.3–44.3] vs. 44.5 [25.8–58.5], *p* = 0.043; SDNN, 79.6 ± 42.6 vs. 99.6 ± 42.3, *p* = 0.004). Other HRV time-domain were also observed to decrease in the CH group, such as RMSSD (59.5 ± 36.9 vs. 77.3 ± 39.4, *p* = 0.077) (Fig. 3). Frequency-domain analysis in the remission period also showed a decreasing trend. However, there were no significant differences between the CH population and the control group (Table 4).

**Table 4:** Parameters of heart rate variability outside the cluster period

	Cluster headache (*n* = 30)	Healthy controls (*n* = 30)	Differences *p* (95% CI)
AV.HR (b/m), median (IQR)	64.2 (59.6–75.8)	60.4 (57.3–62.7)	0.038*
Max.HR (b/m), median (IQR)	76.6 (70.4–85.9)	71.04 (66.1–79.5)	0.143
Min.HR (b/m), median (IQR)	54.0 (46.6–66.1)	49.7 (44.7–58.8)	0.169
E/I Ratio	1.4 (1.3–1.5)	1.5 (1.3–1.7)	0.249
SDNN (ms), mean (SD)	79.6 (42.6)	99.6 (42.6)	0.004*
RMSSD (ms), mean (SD)	59.5 (36.9)	77.3 (39.4)	0.077
pNN50 (%), median (IQR)	31.0 (5.3–44.3)	44.5 (25.8–58.5)	0.043*
LF power (ms^2^), median (IQR)	4454.1 (1618.9–9451.3.9.3)	7657.3 (2636.4–12172.7.4.7)	0.121
HF power (ms^2^), median (IQR)	614.3 (133.0–1645.7.0.7)	1262.2 (307.6–1931.4.6.4)	0.160
LF/HF ratio	7.6 (5.7–14.2)	6.3 (4.4–14.4)	0.492

**Notes**: Values are presented as mean and standard deviation (SD), median and interquartile range (IQR)

**Abbreviations**: AV.HR, average heart rate; Max., maximum; Min., minimum; LF/HF, the ratio of low-frequency/high-frequency power; RMSSD, root mean square of the difference between successive normal intervals; pNN50, the percentage of the number of pairs of consecutive beat-to-beat intervals that differed by 50 ms; SDNN, the standard deviation of the normal-to-normal RR interval; LF, low frequency; HF, high frequency

* *p* < 0.05, significant differences between cluster headache and headache-free controls

### Plasma norepinephrine levels (NE)

**Fig. 4:**
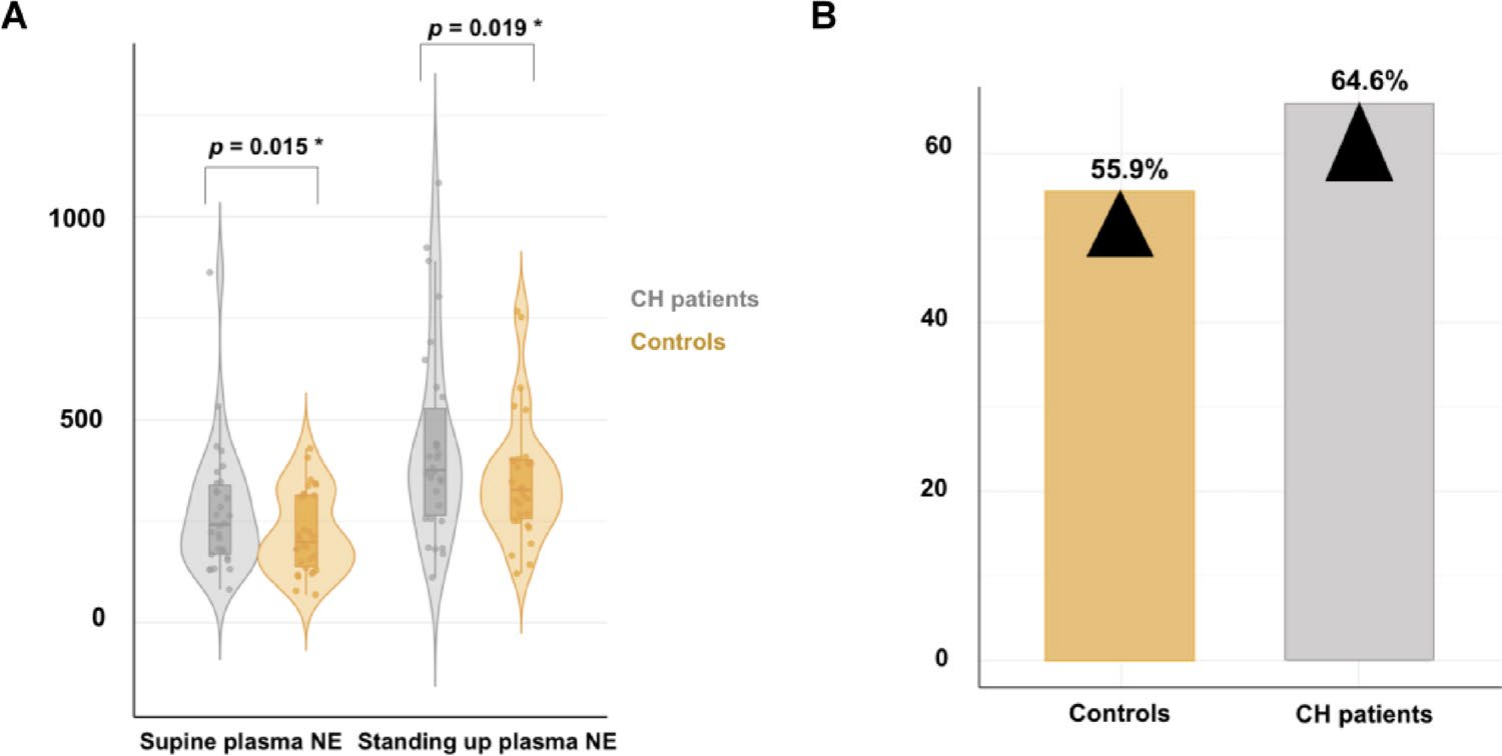
Supine and standing up norepinephrine plasmatic levels in cluster headache (CH) in remission and control group

As shown in Fig. 4, supine and upright NE levels were significantly higher in CH group, 228.9 [161.6–324.1.6.1] pg/mL CH supine and 376.1 [264.6–527.8.6.8] pg/mL standing compared to healthy controls (209.9 [151.2–314.1.2.1] pg/mL and 327.4 [256.4–400.9.4.9] pg/mL, respectively). After standing, plasma NE levels rose by 64.6% of the resting value in the CH group and by 55.9% of the resting value in controls.

Finally, we analysed the correlation between CH- related characteristics and relevant parasympathetic and sympathetic parameters (Table 5). A significant correlation was observed between the reduction in the time-domain HRV parameters (SDNN, RMSSD, PPN50) and a longer duration of CH disease (r_s_ = − 0.923, *p* = 0.018; r_s_ = − 0.836, *p* = 0.039; r_s_ = − 0.909, *p* = 0.022, respectively). A similar correlation was observed with standing up plasma NE levels (r_s_ = − 0.865, *p* = 0.047).

**Table 5:** Correlation analysis results of cluster headache (CH) clinical features that influence on parasympathetic and sympathetic activity

	SDNN		RMSSD		PNN50		Supine plasma NE		Standing up plasma NE		
	**r**_**s**_	***p*** **-value**	**r**_**s**_	***p*** **-value**	**r**_**s**_	***p*** **-value**	**r**_**s**_	***p*** **-value**	**r**_**s**_	***p*** **-value**	
Age of disease onset	0.231	0.220	0.088	0.642	0.135	0.476	0.081	0.671	0.236	0.209	
Disease duration	−0.923	0.018*	−0.836	0.039*	−0.909	0.022*	0.138	0.469	−0.865	0.047*	
Daily attack frequency	0.436	0.016*	0.657	0.335	0.457	0.011*	0.042	0.827	0.356	0.059	
Daily attack duration	0.253	0.178	0.190	0.314	0.157	0.406	0.086	0.651	0.213	0.259	
Number of attacks per year	0.226	0.230	0.277	0.139	0.305	0.101	0,033	0.862	0.114	0.550	
Cluster period duration	0.345	0.179	0.157	0.407	0.115	0.545	0.266	0.155	0.298	0.110	
Last bout	0.655	0.135	0.789	0.046*	0.755	0.045*	0.567	0.078	0.867	0.035	

r_s_: Spearman’s rank correlation coefficient

**Abbreviations**: NE, norepinephrine; pNN50, the percentage of adjacent R-R intervals that differ by more than 50 ms; SDNN, the standard deviation of the RR interval; RMSSD, the root mean square of successive R-R differences

* *p* < 0.05, significant differences between cluster headache and headache-free controls

## Discussion

To the best of our knowledge, this is the first study to comprehensively and homogeneously assess different parameters of autonomic function outside the bout period in CH patients. One of the main findings of this cross-sectional study is that HRV parameters are significantly lower in CH. It looks like that pNN50, RMSSD and SDNN are particularly lower compared to headache-free controls, suggesting an autonomic dysfunction in the time-domain HRV parameters.

HRV reveals the balance in ANS activity, in which an increase in HRV reflects parasympathetic predominance, whereas decreased HRV suggests sympathetic predominance [16]. Numerous studies have investigated HRV abnormalities in various painful conditions, including chronic migraine, in which investigations have consistently demonstrated an autonomic dysfunction as evaluated by HRV [19]. The mechanisms underlying the HRV change patterns in CH during spontaneous attacks confirmed the finding of increased parasympathetic tone [20]. In contrast, investigations of the remission period seem to differ [21, 22].

We demonstrated that out-of-bout CH patients have lower time-domain parameters of HRV (pNN50, RMSSD, and SDNN), in addition to a higher mean HR compared to the control group. Although there were no significant differences between frequency-domain values, we also found a decreasing trend in all parameters evaluated. Our findings suggest a blunted parasympathetic system response in ECH during remission periods.

Similar to our results, some studies revealed differences in time-domain and -frequency parameters in CH. In this regard, Tubani et al., demonstrated severe sympathovagal imbalance during spontaneous attacks and mean LF and HF values during intercritic periods, suggesting ANS dysfunction [21]. Furthermore, an Italian study during spontaneous attacks in 8 CH patients revealed an increase in LF before attacks, followed by an increase in the HF component lasting until the attack subsided [22]. In opposition to these results, the study by van Vliet et al., revealed no systematic cardiovascular autonomic functional changes in CH during a cluster period, outside the pain attack [5]. In contrast to our research, most of the studies focused on monitoring autonomic function during the cluster period. Furthermore, most of the investigations that have evaluated possible changes in remission periods are characterized by their methodological heterogeneity (simple sizes, patients on prophylactic medication, time since last bout unspecified, etc.) [20].

BP and HR values with postural changes are determinant in the evaluation of ANS [23]. However, some patients have a paradoxical increase in upright BP, presumably due to sympathetic activation overshoot. Interestingly, our findings showed an increase at 2 and 3-minutes orthostatic SBP upon active standing in CH patients, compared to headache-free controls. At the same time, supine and 3-minutes supine DBP was higher in CH group. HR was also higher in all phases of the measurements, with a non-significant increasing trend over time, which was not present in healthy controls. Based on our findings, we speculate that there could be an evidence of reduced parasympathetic system and a trend toward increased sympathetic activity with standing during remission periods.

Early observations of BP and HR changes in CH were made during provoked attacks and employing different methodologies in heterogeneous populations [24]. Thus, a Spanish study revealed a higher mean night-time systolic and diastolic BP and non-dipping pattern in CH population; however, the period of the included patients was not specified [25]. The same researchers demonstrated higher carotid intima-media thickness values during remission periods and hypothesized that CH out-of-bout have a higher risk of cardiovascular disease [26]. This is consistent with the orthostatic changes in HR and BP found in our study, in which we homogeneously evaluated patients with CH in the remission phase and without a previous diagnosis of cardiovascular disease.

Plasma NE levels, taken together with the cardiovascular response to tilt and standing up may be a useful index of overall sympathetic function [27]. In our cohort, we observed significant differences in supine and standing plasmatic NE levels between groups. These findings could suggest a possible sympathetic nervous system hyperactivity during pain-free periods in CH.

Some studies have attempted to explore the role of neurotransmitter changes in CH, but, most of them focused on the cluster period and the results are contradictory. Igarashi et al.., observed an increase in NE closely related to the pain attack. Conversely, they found no change in NE by 5 min standing during the remission period, but data for the last bout were not detailed [8]. Stritmatter et al., examined twelve ECH during the cluster period and reported lower plasma and cerebrospinal fluid NE levels compared to controls [10]. Subsequently, a Swedish study observed an altered nocturnal growth hormone pattern in the remission phase, which could indicate a permanent hypothalamic disturbance; despite this, nocturnal secretion of NE, cortisol and insulin did not differ significantly between groups [12]. D’Andrea et al.., were the first to describe the involvement of alpha-agonists in CCH; they found high levels of NE and epinephrine in CCH patients and hypothesized an activation of endothelial receptors trace amine-associated, which may constitute a step in the physiopathology of cluster attacks and a possible cause of chronicity of this primary headache [14, 28]. This could explain the relationship found in our study between elevated NE levels and the duration of the CH disease.

The mechanisms underlying autonomic dysfunction in CH are yet to be fully explored, especially during remission periods. The central autonomic network is responsible for generating headache and CAS in CH. Additionally, more widespread systemic autonomic dysfunction may be present in this disorder [29]. Regarding CH pathophysiology, the hypothalamus role is undeniable in the genesis of attacks. Additional brainstem nuclei -locus coeruleus, raphe, periaqueductal grey- play a role in the regulation of pain input in CH and different cortical-hypothalamic-brainstem functional interconnections can switch between out-of-bout and in-bout periods, igniting the trigeminovascular system [3, 30].

Based on this, some investigations have described the question of whether autonomic symptomatology is also of central origin in CH, and therefore not only present during pain attacks. Barloese et al., performed a narrative review suggesting that interictal subclinical autonomic dysfunction may exist [20]. In this regard, Ofte et al., found a bilateral reduction in cranial parasympathetic tone during remission [7]. These findings support evidence of reduced parasympathetic function in the pain- free state of CH. This is consistent with the results of our study, which suggest a reduction of parasympathetic system activity during remission periods of CH.

Therefore, it could be plausible that the hypothalamus is involved in attacks, but the pathophysiology associated with in-bout/out-of-bout transitions may extend beyond the hypothalamus, and involve dynamic interactions with unidentified cortical and subcortical areas [30]. Recent voxel-based morphometric studies, mainly performed in the absence of pain attacks, have identified structural grey matter changes in mainly areas involved in the pain matrix [31, 32]. These areas are part of the central autonomic network and functional abnormalities in this network could be related to autonomic dysfunction in the remission phase of our CH. Furthermore, white matter microstructural differences have been reported in frontal pain modulation areas during the cluster-bout period and these changes mostly persist during out‐of‐bout periods [33]. Finally, decreased functional co-activation of the hypothalamus and salience network areas has been observed, suggesting association with the defective central pain control pathway and dysregulation of the ANS [34].

Our study has some strengths. First, is the first study to report an integrated autonomic system evaluation in a homogeneous sample of out-bout CH patients. Some previous studies have performed HRV assessment during pain attacks or associated with in-bout/out-of-bout transitions in a heterogeneous sample. We conducted a cross-sectional study to assess sympathetic and parasympathetic activity during remission periods through an accurate and rigorous protocol in all patients. Second, the CH diagnoses of the patients included were well validated, and the recordings were made with standardized methods ensuring at least 12 weeks since the last cluster period and always carried out by the same researchers.

At the same time, the present study has some limitations. First, it is a single-centre study that recruited patients; therefore, we cannot exclude a selection bias. The sample is small, and consequently conclusions should be drawn with caution. In addition, complete matching for smoking was not achieved; smoking is known to influence baroreflex sensitivity, but participants of our study were free of nicotine from the previous evening. Even though, we can not completely rule out that some changes we observed may be attributable to smoking and other lifestyles. Finally, although it is relatively well-established that time and frequency-domain are indexes of autonomic system-mediated HRV, spectral and nonlinear analysis are very sensitive and the conditions under which patients are investigated may be highly influential on the results. Thus, the results should be interpreted with caution and confirmed in a larger study in the future.

## Conclusion

Patients with ECH have reduced time-domain HRV indices and increased plasma NE levels in supine and standing positions, which could reflect a decrease in parasympathetic activity with sympathetic hyperactivity during during pain-free periods. These findings could suggest an imbalance of the ANS and the involvement of different pain-related structures in the physiopathology of CH. Further studies involving more comprehensive and standardized tests of systemic autonomic function are needed in order to improve our knowledge related to dynamic transitioning between in-bout and out-of-bout periods in CH.

## Data Availability

Sharing data compromises ethical standards or legal requirements.

## Abbreviations

ANS: Autonomic nervous system
BP: Blood pressure
CAS: Cranial autonomic symptoms
CH: Cluster headache
CCH: Chronic cluster headache
DBP: Diastolic blood pressure
ECH: Episodic cluster headache
HF: High frequency
HR: Heart rate
IQR: Interquartile range
HRV: Heart rate variability
LF: Low frequency
NE: Norepinephrine
pNN50: Percentage of successive RR intervals
RMSSD: Root mean square of successive RR intervals differences
SDNN: Standard deviation of normal-to-normal RR intervals
SNS: Sympathetic nervous system
